# Immunogenicity and safety of a recombinant COVID-19 vaccine (ZF2001) as heterologous booster after priming with inactivated vaccine in healthy children and adolescents aged 3-17 years: an open-labeled, single-arm clinical trial

**DOI:** 10.1186/s12879-024-09293-1

**Published:** 2024-04-19

**Authors:** Tao Huang, Qianqian Hu, Xiang Zhou, Huaiyu Yang, Wei Xia, Feng Cao, Minglu Deng, Xiaoxue Teng, Fan Ding, Zaixin Zhong, Lidong Gao, Jiufeng Sun, Lihui Gong

**Affiliations:** 1 Hunan Provincial Center for Disease Control and Prevention, Changsha, 410005 China; 2 Anhui Zhifei Longcom Biopharmaceutical, Hefei, 230601 China; 3 Xiangtan Center for Disease Control and Prevention, Xiangtan, 411100 China; 4 Guangdong Provincial Institute of Public Health, Guangzhou, 511430 China

**Keywords:** COVID-2019, SARS-CoV-2, Vaccine, Safety, Immunogenicity, Booster immunization

## Abstract

Considering that neutralizing antibody levels induced by two doses of the inactivated vaccine decreased over time and had fallen to low levels by 6 months, and homologous and heterologous booster immunization programs have been implemented in adults in China. The booster immunization of recombinant COVID-19 vaccine (ZF2001) after priming with inactivated vaccine in healthy children and adolescents has not been reported. We performed an open-labeled, single-arm clinical trial to evaluate the safety and immunogenicity of heterologous booster immunization with ZF2001 after priming with inactivated vaccine among 240 population aged 3-17 years in China. The primary outcome was immunogenicity, including geometric mean titers (GMTs), geometric mean ratios (GMRs) and seroconversion rates of SARS-CoV-2 neutralizing antibodies against prototype SARS-CoV-2 and Omicron BA.2 variant at 14 days after vaccination booster. On day 14 post-booster, a third dose booster of the ZF2001 provided a substantial increase in antibody responses in minors, and the overall occurrence rate of adverse reactions after heterologous vaccination was low and all adverse reactions were mild or moderate. The results showed that the ZF2001 heterologous booster had high immunogenicity and good safety profile in children and adolescents, and can elicit a certain level of neutralizing antibodies against Omicron.

**Trial registration** NCT05895110 (Retrospectively registered, First posted in ClinicalTrials.gov date: 08/06/2023)

**Supplementary Information:**

The online version contains supplementary material available at 10.1186/s12879-024-09293-1.

## Introduction

The massive and rapid transmission of SARS-CoV-2 has led to the emergence of several viral variants of concern (VOCs). From January to June 2022, the dominant SARS-CoV-2 variant globally has been the Omicron variants, with emergence of additional sub-lineages [1]. The Omicron variants showed the highest degree of immune evasion against the current available vaccine compared to other variants [2]. Therefore, more effective vaccination strategies are needed to further control the epidemic trend.

Vaccines based on different tech routes, such as inactivated vaccines, mRNA vaccines, DNA vaccines, viral vector vaccines, and recombinant protein vaccines were applied in human [3]. About 69.1% population of world has received at least one dose of a COVID-19 vaccine as of January 2023 [4].The full vaccination rate of SARS-CoV-2 vaccine had reached as high as 89.7% in China [5]. Considering that most population in China received inactivated vaccines as the primary vaccination and the effectiveness of primary vaccination decreased significantly after 6 months [6], homologous and heterologous booster immunization programs have been implemented in adults in China [7]. As of July 22, 2022, the booster vaccination rate had reached 71.1% in China [5]. If booster immunization is basically achieved in adults, but not in children and adolescents, they may indirectly become susceptible to the virus and increase the risk of disease. WHO and the U.S. CDC have developed vaccination strategies for booster immunization [8, 9], but no recommendations for minors. Previous literature has shown that very low neutralizing antibody GMTs against prototype SARS-CoV-2 and Omicron variants 4-7 months after two doses of inactivated vaccines [10]. In addition, seroconversion levels of neutralizing antibodies against Omicron were significantly improved with the three-dose regimen compared with the two-dose regimen [10]. Previously reported literature has shown that heterologous booster vaccination produces better immune responses than homologous booster vaccination [11–13]. Besides, most studies have been conducted in adults [12, 14–18], and only one study of three-dose CoronaVac homologous immunization was conducted in children and adolescents [19]. A third dose of CoronaVac was safe and elicited robust neutralizing antibody responses to prototype SARS-CoV-2 in children and adolescents and possibly some against the Omicron variant. In addition, a limited number of articles have shown that heterologous booster immunization with a one-dose recombinant protein vaccine is safe and induces high immune responses in adults who have received two doses of an inactivated vaccine [12, 15]. As to now, it is still lacking clinical trial to evaluate the immunogenicity and safety of the heterologous booster vaccination with an inactivated vaccine followed by a recombinant protein vaccine in children and adolescents. More importantly, neutralizing antibodies against the currently circulating Omicron variant were tested in minors of this trial.

## Methods

### Trial design and participants

This open-labeled, single-arm trial was conducted at Xiangtan Center for Disease Control and Prevention in Hunan Province, China. The trial was approved by the Ethics Committee of Hunan Provincial Center for Disease Control and Prevention. The trial was performed in accordance with Declaration of Helsinki and other relevant domestic legal and regulatory requirements. Written informed consent was provided by all participants or their guardians prior to inclusion into the trial.

Healthy children and adolescents aged 3-17 years old, who had previously received two doses of commercially available COVID-19 inactivated vaccine in the past 5-8 months were included in this trial. A total of 240 participants were stratified into three groups: 3-5 years of age (*n*=70), 6-11 years of age (*n*=70), and 12-17 years of age (*n*=100). All participants received one dose of recombinant COVID-19 vaccine (ZF2001). Participants with confirmed or asymptomatic COVID-19 infections or a history of positive nucleic acid testing for COVID-19 or SARS-CoV-2/SARS virus infection were excluded. The details of inclusion and exclusion criteria were listed in trial protocols.

### Procedures

The recombinant COVID-19 vaccine ZF2001 was jointly developed by the Institute of Microbiology, Chinese Academy of Sciences, and Anhui Zhifei Longcom Biopharmaceutical. A recombinant protein vaccine constituting the dimeric SARS-CoV-2 RBD was manufactured according to good manufacturing practice guidelines by Anhui Zhifei Longcom Biopharmaceutical. The vaccine used in the trial were 0.5 mL per vial and contained 25μg recombinant RBD protein and 0.25 mg aluminum hydroxide adjuvant (lot no. D202109134). Vaccines were stored at 2-8°C before use.

All participants underwent axillary temperature measurements at screening, before vaccination and within 7 days after dose. They also underwent physical examination (skin check and Cardiopulmonary auscultation) at screening. For women of childbearing age, urine pregnancy test was performed before enrollment (on the day of inoculation).

The vaccine was administrated to eligible participants. After intramuscular administration in the arm, participants were observed in the observation room for at least 30 minutes while all solicited and unsolicited adverse events were recorded and assessed by the investigator. During the first 7 days after vaccination, any solicited and unsolicited adverse events were self-reported by participants or their guardians daily on the diary cards. On postvaccination day 8, the diary cards were retrieved by the investigator to assess all adverse events. Unsolicited adverse events occurring 8-30 days after vaccination were reported by participants or their guardians through contact cards. The contact cards were retrieved on postvaccination day 31.

All adverse events included all solicited and unsolicited adverse events at 30 minutes, solicited and unsolicited adverse events between 0-7 days, and unsolicited adverse events between 8-30 days. All adverse events between 0-30 days that were assessed by the investigator whether they were related to the vaccine. Only adverse events related to vaccination were defined as adverse reactions.

Solicited local adverse events included injection site pain, swelling, induration, redness, rash, itching, and cellulitis. Solicited systemic adverse events after vaccination included fever, headache, diarrhea, vomiting, acute allergic reactions, cough, nausea, muscle pain (not at the injection site), fatigue, and adynamia. The criteria for grading adverse events and adverse reactions were based on the Guidelines for Grading Adverse reactions in Clinical Trials of Vaccines for Preventive Use issued by the NMPA (No.102, 2019). The safety data reported here were from April 29, 2022 to July 9, 2022.

About 5mL blood samples of all participants were collected before vaccination and 14 days after vaccination and sent to the Guangdong Provincial Institute of Public Health for neutralizing antibody detection by Cytopathic methods to assess the neutralizing activity against SARS-CoV-2.

### Outcomes

For the trial, the primary outcome was the immunogenicity of the COVID-19 vaccine. The secondary outcome was safety. The analyses evaluated immunogenicity outcomes by humoral immune responses at 14 days after vaccination and safety outcomes by adverse events at 30 days.

The immunogenicity outcomes were seroconversion rate and magnitude, in geometric mean titers (GMTs), of SARS-CoV-2 neutralizing antibodies against SARS-CoV-2 prototype and Omicron BA.2 variant. Seroconversion rate was defined as the percentage of participants with either a pre-vaccination neutralizing antibody titer <1:4 and a post-vaccination neutralizing antibody titer ≥1:4, or a pre-vaccination neutralizing antibody titer ≥1:4 and a ≥4 folds increase in post-vaccination neutralizing antibody titer.

Safety outcomes included: all adverse events within 30 days after vaccination (including all adverse events at 30 minutes, all solicited and unsolicited adverse events at 0-7 days, and all unsolicited adverse events at 8-30 days), adverse events related to the vaccine, adverse events of grade 3 and worse, and adverse events leading to withdrawal of participants, serious adverse events.

### Statistical analysis

The exploratory trial did not calculate the target sample size based on statistical power, 240 participants were recruited: 3-5 years old (*n*=70), 6-11 years old (*n*=70), and 12 -17 years old (*n*=100).

Immunogenicity was analyzed for the per-protocol set (PPS) and the full analysis set (FAS), with the PPS as the primary set as defined in the study protocol. The PPS refers to participants who adhered to the inclusion and exclusion criteria and the trial protocol, who completed the booster vaccination and had valid immunogenicity results both pre-immunization and 14 days after immunization. The FAS population comprised all participants who received at least one vaccination and had a valid pre-immunization immunogenicity result. The safety analysis was performed for the safety set (SS), which consisted of all participants that were vaccinated.

Neutralizing antibody titer against the SARS-CoV-2 prototype and the Omicron BA.2 variant were summarized by means of GMTs, GMRs (post-vaccination versus pre-vaccination) and seroconversion rates. The Clopper-Pearson method was used to calculate 95% confidence intervals (CIs) for the seroconversion rates. The safety analysis was limited to the reporting of numbers and proportions of participants with adverse events or serious adverse events after vaccination. SAS software (version 9.4) was used for the statistical analysis.

## Results

**Figure 1.**
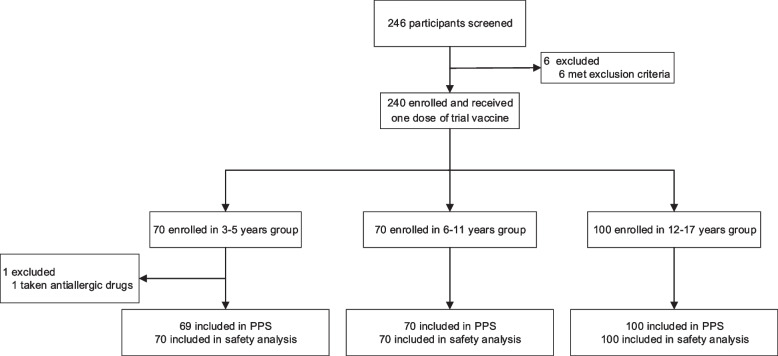
Trial profile. Eligible participants who had previously received two doses of commercially available COVID-19 inactivated vaccine in the past 5-8 months were enrolled to receive one dose of recombinant COVID-19 vaccine (ZF2001). A total of 240 participants received one dose of ZF2001. All participants were included in the full analysis set (FAS) and the safety set (SS). One participant was excluded from the per-protocol set (PPS) because of a protocol violation due to the use of an allergic medication

Between April 29 and May 6, 2022, 246 children and adolescents were recruited for eligibility in Xiangtan, China. After exclusion of 6 individuals, 240 participants, including 70 aged 3-5 years, 70 aged 6-11 years, and 100 aged 12-17 years, received one dose of 25μg ZF2001 and were included in the SS and the FAS, and all completed the follow-up visit on day 14. A total of 239 participants were included in PPS. The participant was excluded from PPS because of a protocol violation due to the use of an allergic medication (Fig. 1). The mean age of all participants was 9.6 years (SD 4.2) (table 1).

**Table 1:** Baseline demographic characteristics of participants

	**3-5 years (*n*=70)**	**6-11 years (*n*=70)**	**12-17years (*n*=100)**	**3-17 years (*n*=240)**
**Age (years)**				
Mean (SD)	4.4(0.7)	8.7(1.4)	13.9(1.3)	9.6(4.2)
Median (IQR)	4.0(4.0,5.0)	9.0(8.0,10.0)	14.0(13.0,15.0)	10.0(5.0,13.0)
Min-Max	3.0-5.0	6.0-11.0	12.0-17.0	3.0-17.0
**Height (cm)**				
Mean (SD)	110.8(5.9)	135.0(11.0)	164.2(9.0)	140.1(24.1)
Median (IQR)	111.3(107.0,114.5)	136.0(128.0,141.0)	163.5(157.5,171.0)	139.3(115.0,161.0)
Min-Max	98.5-124.0	108.0-160.0	141.0-186.0	98.5-186.0
**Weight (kg)**				
Mean (SD)	18.3(2.2)	31.4(10.1)	54.6(12.7)	37.2(18.4)
Median (IQR)	17.8(16.9,19.4)	28.5(25.1,35.1)	52.6(46.7,59.7)	32.5(20.1,51.2)
Min-Max	13.5-27.4	17.5-73.9	28.8-94.5	13.5-94.5
**Gender**				
Male	36(51.4)	32(45.7)	58(58.0)	126(52.5)
Female	34(48.6)	38(54.3)	42(42.0)	114(47.5)
**Ethnicity**				
Han Chinese	68(97.1%)	69(98.6%)	99(99.0%)	236(98.3%)
Other	2(2.9%)	1(1.4%)	1(1.0%)	4(1.7%)

**Figure 2.**
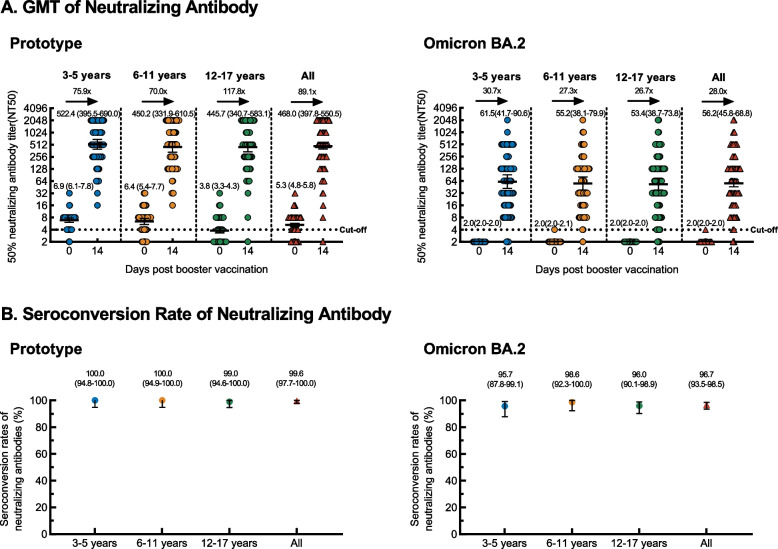
The GMTs and Seroconversion rates of neutralizing antibodies against SARS-CoV-2 Prototype and Omicron BA.2 (PPS). For (A) GMTs of neutralizing antibodies in stratified age groups (3-5 years, 6-11 years and 12-17 years of age) and in all participants before booster vaccination (day 0) and after a booster dose of ZF2001 (day 14). (B) Seroconversion rates of neutralizing antibodies in different age subgroups (3-5 years, 6-11 years and 12-17 years of age) and in all participants after a booster dose of ZF2001 (day 14). GMTs, geometric mean titers. GMRs, geometric mean ratios (day 14 vs day 0). Seroconversion rate was defined as the percentage of participants with either a pre-vaccination neutralizing antibody titer <1:4 and a post-vaccination neutralizing antibody titer ≥1:4, or a pre-vaccination neutralizing antibody titer ≥1:4 and a ≥4 folds increase in post-vaccination neutralizing antibody titer. Numbers in parentheses are 95% confidence intervals (95% CIs). Error bars represent 95% CIs. Arrows represent GMRs.

The baselines of the neutralizing antibodies against SARS-CoV-2 prototype and Omicron BA.2 variant are shown in Appendix Table S1. On day 14 after vaccination, the seroconversion rate of neutralizing antibodies against prototype SARS-CoV-2 in population aged 3-17 years was 99.6% (238 of 239) with GMTs of 468.0 (95% CI 397.8-550.5) (Fig. 2A, B, Appendix Table S2). The GMTs of stratified 3-5-, 6-11-, and 12-17-years age groups were 522.4 (95% CI 395.5-690.0), 450.2 (95% CI 331.9-610.5) and 445.7 (95% CI 340.7-583.1), respectively (Fig. 2A, Appendix Table S2). For participants receiving the heterologous booster with ZF2001, neutralizing antibodies GMTs increased from baseline by 89.1 folds (95%CI 76.1-104.2) in population aged 3-17 years. GMTs increased 75.9 folds (95%CI 57.9-99.6), 70.0 folds (52.9-92.6), and 117.8 folds (91.1-152.2) in the three groups at different ages, respectively (Fig. 2A, Appendix Table S2). However, the seroconversion rate of neutralizing antibodies against SARS-CoV-2 Omicron BA.2 variant decreased to 96.7% (231 of 239) in population aged 3-17 years with GMTs of 56.2 (95% CI 45.8-68.8) (Fig. 2A, B, Appendix Table S2). The GMTs of stratified 3-5-, 6-11-, and 12–17-year-old groups were 61.5 (95% CI 41.7-90.6), 55.2 (95% CI 38.1-79.9), and 53.4 (95% CI 38.7-73.8), respectively (Fig. 2A, Appendix Table S2). For participants receiving the heterologous booster with ZF2001, neutralizing antibodies GMTs increased from baseline by 28.0 folds (95%CI 22.8-34.3) in population aged 3-17 years. GMTs increased 30.7 folds (95%CI 20.8-45.3), 27.3 folds (18.8-39.6), and 26.7 folds (19.4-36.9) in the three groups at different ages, respectively (Fig. 2A, Appendix Table S2).

**Figure 3.**
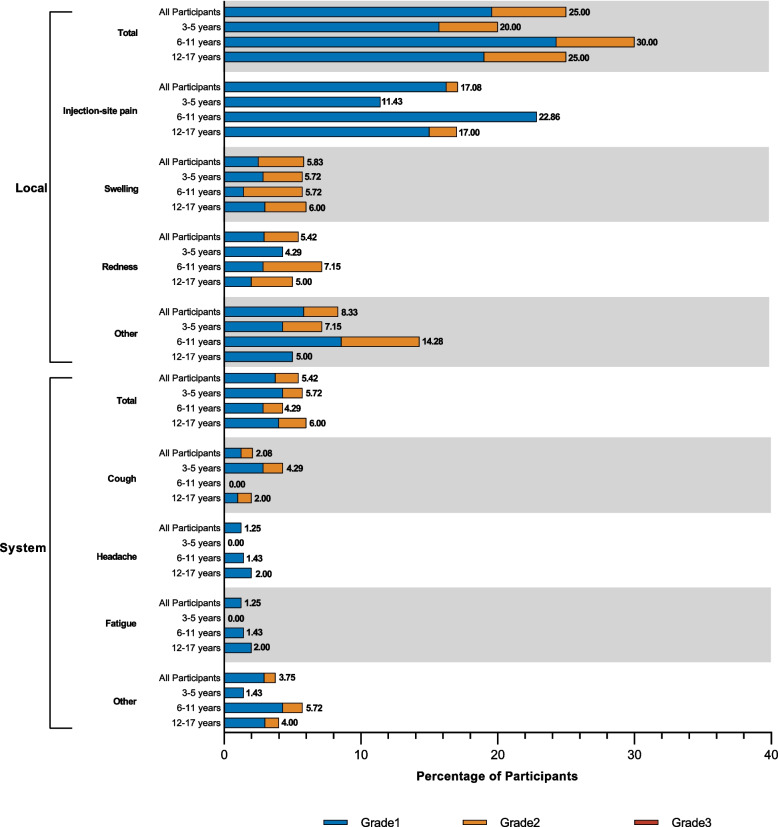
Solicited local and systemic adverse reactions graded according to severity in different age groups. Shown are the percentages of participants in whom solicited local or systemic adverse reactions occurred within 30 days after a booster dose of ZF2001 in the safety set (SS). The age groups observed included 3-5 years, 6-11 years, 12-17 years and 3-17 years of age (all participants)

In this trial, adverse events within 30 days after vaccination were recorded in 31.7% (76 of 240) of healthy children and adolescents aged 3-17 years. The incidence of adverse reactions within 30 days after vaccination was 23.8% (57 of 240). 13 (18.6%) of 70 participants aged 3-5 years, 19 (27.1%) of 70 participants aged 6-11 years, and 25 (25.0%) of 100 participants aged 12-17 years reported at least one adverse reaction within 30 days after vaccination. The incidences of adverse reactions in the three age groups was close to the overall incidence. 21.7% (52 of 240) of the participants reported solicited local adverse reactions. The most common reactions were injection-site pain, swelling and redness. The most common solicited systemic adverse reactions were cough, fever, and headache with an incidence of 5.0% (12 of 240). There were no unsolicited adverse reactions. The majority adverse events and adverse reactions occurred within 7 days after vaccination. Within 30 days after vaccination, all the adverse reactions were mild or moderate (grade 1 or 2), and no grade 3 or above adverse reactions were observed. Two serious adverse events (cellulitis and allergic dermatitis) from one participant were noted, but none was considered to be related to the study vaccine as assessed by the investigators. There were no adverse reactions that caused participants to drop out. We have monitored the risk of antibody dependent enhancement (ADE) caused by vaccine since the beginning of the trial, and none of the participants was diagnosed as COVID-19 and no ADE case was reported. Details of these safety data are provided in Fig. 3 and Appendix Table S3.

## Discussion

This trial revealed the immunogenicity and safety of a booster dose with a recombinant COVID-19 vaccine (ZF2001) in population aged 3-17 years. To our knowledge, this is the first reported result of heterologous booster with the recombinant protein vaccine in children and adolescents who received inactivated vaccines. The results showed that the ZF2001 heterologous booster had high immunogenicity and good safety profile in children and adolescents. Considering that inactivated vaccines has been applied in large-scale populations and the ZF2001 booster vaccination can elicit a certain level of neutralizing antibodies against Omicron. Therefore, further studies on heterologous booster immunization in minors are valuable for the prevention of COVID-19 pandemic.

Neutralizing antibody levels induced by two doses of the inactivated vaccine decreased over time and had fallen to low levels by 6 months [6]. The results of our study showed that preimmunization antibody titers in minors enrolled in this study were similarly low. On day 14 post-booster, a third dose booster of the ZF2001 provided a substantial increase in antibody responses against prototype SARS-CoV-2 after two doses of inactivated vaccines in minors, which was similar to the result of the homologous booster study in minors [19]. Of course, we observed a similar increasing trend in adults [12, 14–18]. In the analysis of age subgroups, we observed differences in neutralizing antibody levels by age. The 3-5-year-old group produced higher levels of antibodies than the 6-11- and 12–17-year-old groups. A recently published article speculates that this phenomenon was related to the degradation and atrophy of the thymus during the onset of puberty leading to worse humoral immunity [20].Our study also found that the GMTs of neutralizing antibodies against the prototype SARS-CoV-2 increased 89.1 folds from baseline, which was much higher than the 33.9 folds increase in adults in other study [21]. The results showed that the immune response induced by booster dose in minors was stronger than that in adults. Similar results were also observed in trials of recombinant adenovirus type-5-vectored vaccine and inactivated vaccines [19, 22].

Although the neutralizing antibody titers against Omicron BA.2 variant increased, it was lower than that against the prototype SARS-CoV-2, which was also consistent with the findings in the other reports [16, 19]. Omicron is more likely to escape vaccine-induced immune protection than the prototypes. However, there is increasing evidence that three doses of booster vaccination elicit significantly higher neutralizing titers against the Omicron variants compared to two-dose vaccinations [16, 23]. Our results also implied that heterologous booster immunization might improve neutralization against Omicron. Multivalent vaccines against Omicron subvariants should also be developed as soon as possible to cope with the evolving variant subtypes. The heterologous booster combinations among viral vector vaccines, inactivated vaccines, and mRNA vaccines had been proved to significantly improved humoral immune responses, with heterologous booster having better immunogenicity than homologous booster [13, 24]. There were several trials regarding heterologous booster of recombinant protein vaccines in adults [12, 14–18]and only one trial of three-dose CoronaVac homologous immunization in children and adolescents [19]. Our findings supported that the heterologous booster of recombinant protein vaccines was effective against SARS-CoV-2 in children and adolescents.

For safety, the overall occurrence rate of adverse reactions after heterologous vaccination was low and all adverse reactions were mild or moderate, similar to previous studies of recombinant protein vaccines booster immunization with inactivated vaccines in adults [12, 15]. A booster dose of ZF2001 did not significantly increase the risk of more serious adverse reactions, which was also comparable to the safety of the priming with two doses of inactivated vaccine in children and adolescents [25–27]. There was no obvious difference in overall safety between this recombinant protein vaccines heterologous booster and the homologous booster [12, 15]. However, the occurrence rate of adverse reactions of viral vector vaccines and mRNA vaccines was slightly higher than recombinant protein vaccines as heterologous booster [28–31]. ZF2001 was safe for heterologous booster immunization in minors.

There were several limitations in this study. First, data on immune persistence of the booster vaccination is not available, and the long-term immunogenicity needs to be further studied. Second, our study was a single-arm trial, so further studies are needed to evaluate the immunogenicity and safety of different types of booster vaccines in minors. Third, given the limited sample size and Han Chinese population in our study, larger studies need to be carried out to expand the applicability of this booster strategy. Finally, with the constant variation of COVID-19, some highly transmitted VOC (Variants of Concern) mutants have shown changes in pathogenicity and transmission characteristics, resulting in a new wave of infection worldwide. ZF2001 is a recombinant subunit protein vaccine developed only for the COVID-19 prototype strain, which can no longer cope with COVID-19 infection caused by the new variant strain, so we will continue to develop an improved vaccine for the epidemic strain.

## Conclusions

One dose of heterologous booster vaccination of ZF2001 after priming vaccination of two-dose inactivated vaccines in children and adolescents aged 3-17 years had satisfying safety and immunogenicity. Our findings support the ZF2001 approved for heterologous booster vaccination against COVID-19 in the future.

### Supplementary Information

**Supplementary Material 1.** 

## Data Availability

The datasets used and/or analyzed during the current study are available from the corresponding author on reasonable request.
