# Supplementary material for: Immunogenicity and safety of a recombinant COVID-19 vaccine (ZF2001) as heterologous booster after priming with inactivated vaccine in healthy children and adolescents aged 3-17 years: an open-labeled, single-arm clinical trial
Source: BMC Infect Dis. 2024 Apr 19;24:413. doi: 10.1186/s12879-024-09293-1 (PMC11027523; doi:10.1186/s12879-024-09293-1)
Supplement: Supplementary file 1 — Supplementary Material 1. [file 12879_2024_9293_MOESM1_ESM.docx]

Appendix

**Content**

[Table S1. The neutralizing antibodies in sera at baseline 2](#_Toc161663075)

[Table S2. The neutralizing antibodies on 14 days after immunization 3](#_Toc161663076)

[Figure S1. The GMTs and Seroconversion rates of neutralizing antibodies for SARS-CoV-2 Prototype and Omicron BA.2 (FAS) 4](#_Toc161663077)

[Table S3. Adverse events and adverse reactions after vaccination in different age groups 5](#_Toc161663078)

## Table S1. The neutralizing antibodies in sera at baseline

| **PPS** | **3-5 years**  **(n=69)** | **6-11 years**  **(n=70)** | **12-17 years**  **(n=100)** | **3-17 years**  **(n=239)** |
| --- | --- | --- | --- | --- |
| **Neutralizing antibodies to prototype SARS-CoV-2** | | | | |
| **≥ detection limit (1:4)** | 66 | 58 | 53 | 177 |
| **GMT (95% CI)** | 6.9(6.1,7.8) | 6.4(5.4,7.7) | 3.8(3.3,4.3) | 5.3(4.8,5.8) |
| **Neutralizing antibodies to Omicron** | | | | |
| **≥ detection limit (1:4)** | 0 | 1 | 0 | 1 |
| **GMT (95% CI)** | 2.0(2.0,2.0) | 2.0(2.0,2.1) | 2.0(2.0,2.0) | 2.0(2.0,2.0) |
| **FAS** | **3-5 years**  **(n=70)** | **6-11 years**  **(n=70)** | **12-17 years**  **(n=100)** | **3-17 years**  **(n=240)** |
| **Neutralizing antibodies to prototype SARS-CoV-2** | | | | |
| **≥ detection limit (1:4)** | 67 | 58 | 53 | 178 |
| **GMT (95% CI)** | 6.8(6.0,7.7) | 6.4(5.4,7.7) | 3.8(3.3,4.3) | 5.2(4.8,5.8) |
| **Neutralizing antibodies to Omicron** | | | | |
| **≥ detection limit (1:4)** | 0 | 1 | 0 | 1 |
| **GMT (95% CI)** | 2.0(2.0,2.0) | 2.0(2.0,2.1) | 2.0(2.0,2.0) | 2.0(2.0,2.0) |

GMT, geometric mean titer

## Table S2. The neutralizing antibodies on 14 days after immunization

| **PPS** | **3-5 years**  **(n=69)** | **6-11 years**  **(n=70)** | **12-17years**  **(n=100)** | **3-17 years**  **(n=239)** |
| --- | --- | --- | --- | --- |
| **Neutralizing antibodies to prototype SARS-CoV-2** | | | | |
| **Seroconversion, rate (95% CI)** | 69,100.0%(94.8,100.0) | 70,100.0%(94.9,100.0) | 99,99.0%(94.6,100.0) | 238,99.6%(97.7,100.0) |
| **GMT (95% CI)** | 522.4(395.5,690.0) | 450.2(331.9,610.5) | 445.7(340.7,583.1) | 468.0(397.8,550.5) |
| **GMR (95% CI)** | 75.9(57.9,99.6) | 70.0(52.9,92.6) | 117.8(91.1,152.2) | 89.1(76.1,104.2) |
| **Neutralizing antibodies to Omicron** | | | | |
| **Seroconversion, rate (95% CI)** | 66,95.7%(87.8,99.1) | 69,98.6%(92.3,100.0) | 96,96.0%(90.1,98.9) | 231,96.7%(93.5,98.5) |
| **GMT (95% CI)** | 61.5(41.7,90.6) | 55.2(38.1,79.9) | 53.4(38.7,73.8) | 56.2(45.8,68.8) |
| **GMR (95% CI)** | 30.7(20.8,45.3) | 27.3(18.8,39.6) | 26.7(19.4,36.9) | 28.0(22.8,34.3) |
| **FAS** | **3-5 years**  **(n=70)** | **6-11 years**  **(n=70)** | **12-17years**  **(n=100)** | **3-17 years**  **(n=240)** |
| **Neutralizing antibodies to prototype SARS-CoV-2** | | | | |
| **Seroconversion, rate (95% CI)** | 70,100.0%(94.9,100.0) | 70,100.0%(94.9,100.0) | 99,99.0%(94.6,100.0) | 239,99.6%(97.7,100.0) |
| **GMT (95% CI)** | 527.4(400.7,694.3) | 450.2(331.9,610.5) | 445.7(340.7,583.1) | 469.5(399.3,552.0) |
| **GMR (95% CI)** | 77.2(59.0,101.2) | 70.0(52.9,92.6) | 117.8(91.1,152.2) | 89.5(76.5,104.6) |
| **Neutralizing antibodies to Omicron** | | | | |
| **Seroconversion, rate (95% CI)** | 67,95.7%(88.0,99.1) | 69,98.6%(92.3,100.0) | 96,96.0%(90.1,98.9) | 232,96.7%(93.5,98.6) |
| **GMT (95% CI)** | 62.1(42.4,91.1) | 55.2(38.1,79.9) | 53.4(38.7,73.8) | 56.4(46.0,69.0) |
| **GMR (95% CI)** | 31.1(21.2,45.6) | 27.3(18.8,39.6) | 26.7(19.4,36.9) | 28.1(22.9,34.4) |

GMT, geometric mean titer; GMR, geometric mean ratio on Day 14 vs Day 0; Seroconversion rate: The percentage of participants with either a pre-vaccination neutralizing antibody titer <1:4 and a post-vaccination neutralizing antibody titer ≥1:4, or a pre-vaccination neutralizing antibody titer ≥1:4 and a ≥4 folds increase in post-vaccination neutralizing antibody titer.

## Figure S1. The GMTs and Seroconversion rates of neutralizing antibodies for SARS-CoV-2 Prototype and Omicron BA.2 (FAS)


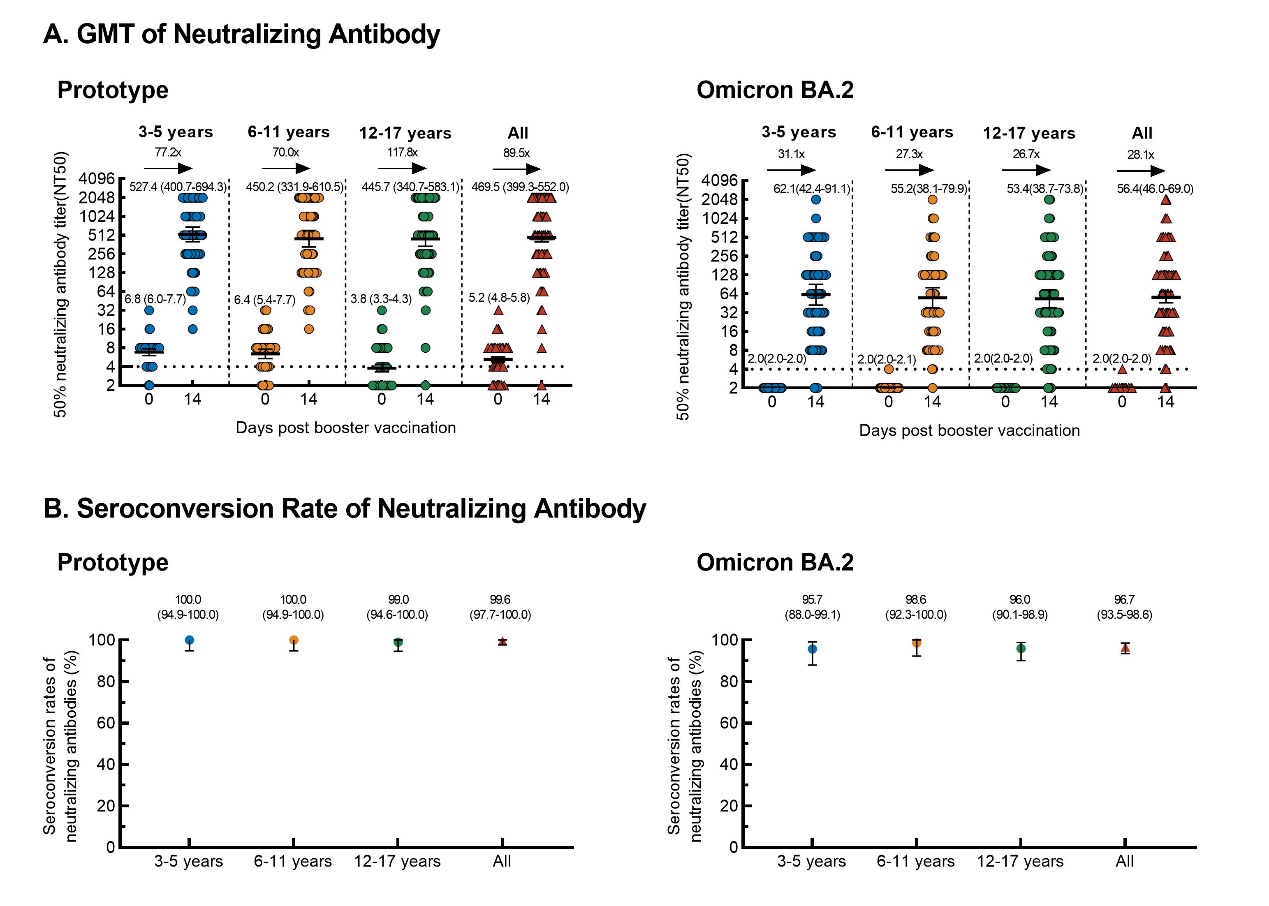


For (A) GMTs of neutralizing antibodies in different age subgroups (3-5 years, 6-11 years and 12-17 years of age) and in all participants before booster vaccination (day 0) and after a booster dose of ZF2001 (day 14). (B) Seroconversion rates of neutralizing antibodies in stratified age groups (3-5 years, 6-11 years and 12-17 years of age) and in all participants after a booster dose of ZF2001 (day 14). GMTs, geometric mean titers. GMRs, geometric mean ratios (day 14 vs day 0). Seroconversion rate was defined as the percentage of participants with either a pre-vaccination neutralizing antibody titer <1:4 and a post-vaccination neutralizing antibody titer ≥1:4, or a pre-vaccination neutralizing antibody titer ≥1:4 and a ≥4 folds increase in post-vaccination neutralizing antibody titer. Numbers in parentheses are 95% confidence intervals (95% CIs). Error bars represent 95% CIs. Arrows represent GMRs.

## Table S3. Adverse events and adverse reactions after vaccination in different age groups

|  | **3-5 years(n=70)** | | **6-11 years(n=70)** | | **12-17 years(n=100)** | | **3-17 years (n=240)** | |
| --- | --- | --- | --- | --- | --- | --- | --- | --- |
|  | **Events** | **Subjects** | **Events** | **Subjects** | **Events** | **Subjects** | **Events** | **Subjects** |
|  | **n** | **n (%)** | **n** | **n (%)** | **n** | **n (%)** | **n** | **n (%)** |
| **Overall AEs within 30days** |  |  |  |  |  |  |  |  |
| Any | 35 | 21(30.0%) | 52 | 22(31.4%) | 54 | 33(33.0%) | 141 | 76(31.7%) |
| Grade ≥3 | 2 | 1(1.4%) | 0 | 0 | 0 | 0 | 2 | 1(0.4%) |
| **ARs within 30days** |  |  |  |  |  |  |  |  |
| Any | 24 | 13(18.6%) | 42 | 19(27.1%) | 45 | 25(25.0%) | 111 | 57(23.8%) |
| ARs within 30min | 1 | 1(1.4) | 4 | 3(4.3) | 6 | 3(3.0) | 11 | 7(2.9) |
| ARs within day 0-7 | 24 | 13(18.6%) | 42 | 19(27.1%) | 45 | 25(25.0%) | 111 | 57(23.8%) |
| ARs within day 8-30 | 0 | 0 | 0 | 0 | 0 | 0 | 0 | 0 |
| Grade 1 | 19 | 13(18.6) | 31 | 18(25.7) | 35 | 21(21.0) | 85 | 52(21.7) |
| Grade 2 | 5 | 4(5.7) | 11 | 5(7.1) | 10 | 8(8.0) | 26 | 17(7.1) |
| Grade ≥3 | 0 | 0 | 0 | 0 | 0 | 0 | 0 | 0 |
| **Solicited ARs** |  |  |  |  |  |  |  |  |
| Any | 24 | 13(18.6%) | 41 | 19(27.1%) | 43 | 25 (25.0%) | 108 | 57(23.8%) |
| **Solicited systemic ARs** |  |  |  |  |  |  |  |  |
| Any | 4 | 4(5.7%) | 6 | 2(2.9%) | 10 | 6(6.0%) | 20 | 12(5.0%) |
| Cough | 3 | 3(4.3%) | 0 | 0 | 2 | 2(2.0%) | 5 | 5(2.1%) |
| Fever | 1 | 1(1.4%) | 2 | 2(2.9%) | 1 | 1(1.0%) | 4 | 4 (1.7%) |
| Headache | 0 | 0 | 1 | 1(1.4%) | 2 | 2(2.0%) | 3 | 3(1.3%) |
| Fatigue | 0 | 0 | 1 | 1(1.4%) | 2 | 2(2.0%) | 3 | 3(1.3%) |
| Nausea | 0 | 0 | 1 | 1(1.4%) | 1 | 1(1.0%) | 2 | 2(0.8%) |
| Diarrhea | 0 | 0 | 0 | 0 | 1 | 1(1.0%) | 1 | 1(0.4%) |
| Vomiting | 0 | 0 | 1 | 1(1.4%) | 0 | 0 | 1 | 1(0.4%) |
| Muscle pain | 0 | 0 | 0 | 0 | 1 | 1(1.0%) | 1 | 1(0.4%) |
| Acute allergic reaction | 0 | 0 | 0 | 0 | 0 | 0 | 0 | 0 |
| **Solicited local ARs** |  |  |  |  |  |  |  |  |
| Any | 20 | 12(17.1%) | 35 | 18(25.7%) | 33 | 22(22.0%) | 88 | 52(21.7%) |
| Injection-site pain | 8 | 8(11.4%) | 16 | 16(22.9%) | 17 | 17(17.0%) | 41 | 41(17.1%) |
| Swelling | 4 | 4(5.7%) | 4 | 4(5.7%) | 6 | 6(6.0%) | 14 | 14(5.8%) |
| Redness | 3 | 3(4.3%) | 5 | 5(7.1%) | 5 | 5(5.0%) | 13 | 13(5.4%) |
| Itch | 2 | 2(2.9%) | 4 | 4(5.7%) | 3 | 3(3.0%) | 9 | 9(3.8%) |
| Induration | 2 | 2(2.9%) | 4 | 4(5.7%) | 2 | 2(2.0%) | 8 | 8(3.3%) |
| Rash | 1 | 1(1.4%) | 2 | 2(2.9%) | 0 | 0 | 3 | 3(1.3%) |
| Cellulitis | 0 | 0 | 0 | 0 | 0 | 0 | 0 | 0 |
| **Unsolicited ARs** | 0 | 0 | 0 | 0 | 0 | 0 | 0 | 0 |
| **Unrelated AEs within 30days** |  |  |  |  |  |  |  |  |
| Any | 11 | 10(14.3%) | 10 | 8(11.4%) | 9 | 9(9.0%) | 30 | 27(11.3%) |
| Grade ≥3 | 2 | 1(1.4%) | 0 | 0 | 0 | 0 | 2 | 1(0.4%) |
| **AEs caused quit** | 0 | 0 | 0 | 0 | 0 | 0 | 0 | 0 |


Table S3 shows the adverse events and adverse reactions that occurred within 30 days after a booster dose of ZF2001 in the safety set (SS). AE: Adverse events; AR: adverse reactions, AEs related to the study vaccine were defined as ARs, whether solicited or unsolicited AEs.
